# Value of susceptibility-weighted imaging for the assessment of angle measurements reflecting hip morphology

**DOI:** 10.1038/s41598-020-77671-1

**Published:** 2020-12-01

**Authors:** Sarah M. Böker, Lisa C. Adams, Ute Lina Fahlenkamp, Gerd Diederichs, Bernd Hamm, Marcus R. Makowski

**Affiliations:** 1 Department of Radiology, Charité, Charitéplatz 1, 10117 Berlin, Germany; 2 Department of Radiology, Technical University of Munich, Ismaninger Str. 22, 81675 Munich, Germany; 3 King’s College London, School of Biomedical Engineering and Imaging Sciences, United Kingdom, St Thomas’ Hospital Westminster Bridge Road, SE1 7EH London, UK

**Keywords:** Bone, Skeleton, Signs and symptoms

## Abstract

Radiographs are the clinical first line imaging modality for evaluating hip morphology and pathology. MRI offers additional information and is the method of choice to evaluate soft tissue, bone marrow and preradiographic signs of osteoarthritis. Radiographs are used to measure the most morphometric parameters. The aim of this study was to compare susceptibility weighted MRI (SWMR) with radiographs to evaluate hip morphology. 40 Patients were examined with standard MR-sequences, coronal SWMR and radiographs in anteroposterior pelvic view. Coronal maximum intensity projection (MIP) images of both hips were automatically reconstructed on SWMR and T1weighted images. Sharp´s angle, Tönnis angle, lateral center–edge angle of Wiberg and caput-collum-diaphyseal angle were measured on coronal SWMR MIP-images, T1weighted MIP-images and radiographs. Measurements were compared by linear regression analysis and Bland-Altmann Plots, using radiographs as reference standard. Additionally, a ratio between the signal intensity of muscles and bone on SWMR and T1weighted MIP-images was calculated and compared between these two sequences. SWMR enables the reliable assessment of Sharp´s angle (SWMR: R^2^ = 0.80; T1weighted: R^2^ = 0.37), Tönnis angle (SWMR: R^2^ = 0.86; T1weighted: not measurable), lateral center–edge angle of Wiberg (SWMR: R^2^ = 0.88; T1weighted: R^2^ = 0.40) and caput-collum-diaphyseal angle (SWMR: R^2^ = 0.38; T1weighted: R^2^ = 0.18) compared to radiographs with a higher accuracy than conventional MR imaging. The ratio between the intensity of muscles and bone was significant higher on SWMR (2.00 and 2.02) than on T1weighted MIP-images (1.6 and 1.42; p < 0.001).

## Introduction

Magnetic resonance imaging, radiographs, computed tomography and ultrasound are the most frequent imaging modalities used in clinical practice to assess pathologies and deformities of the hip. MRI has a high soft tissue contrast and a high sensitivity for bone marrow pathologies^1^. It is the method of choice to evaluate chondral lesions, pathologies of the acetabular labrum and the peri-articular soft tissue. A limitation of conventional MRI is the visualisation of calcified structures such as the bone matrix.

In adults, MRI plays an important role in the assessment of osteoarthritis. Causes leading to early osteoarthritis include deformities of the acetabulum or the femur like dysplastic hips or CAM/FAI impingement^2,3^. To quantify and confirm the visual impression of the deformity, morphometric parameters are measured.

In clinical practice, morphometric parameters to assess hip deformity such as the lateral center–edge angle of Wiberg, Tönnis angle, Sharp´s angle and the caput-collum-diaphyseal angle are measured on radiographic images, e.g. the anteroposterior (AP) pelvic view. Specific protocols can also include additional other projections like frog leg or lateral views. The lateral center–edge angle of Wiberg is an angle to quantify the acetabular coverage^4^. To assess the acetabular inclination, Tönnis angle and Sharp´s angle are determined^1,5^. The caput-collum-diaphyseal angle assesses the longitudinal axis between the femoral neck and shaft^6^. Reference values for the above mentioned angles relate to 2 dimensional radiographs^7^, not for 3 dimensional imaging methods like MRI. However, radiographs are limited by their failure to appreciate the three dimensional morphometry of the joint and the use of ionizing radiation. In recent years the above mentioned measures have been validated using three dimensional imaging platforms such as MRI^8,9^. MRI offers better evaluation of the shape of the joint and the soft tissue, however standard protocols struggle to adequately assess bone structure. Susceptibility weighted MRI (SWMR) allows morphological assessment of bone structures without the need for ionizing radiation and could be therefore be a useful clinically sequence. This would especially be relevant for repetitive examinations in young patients.

SWMR is a 3D gradient-echo(GRE)-technique, which can visualise calcified structures like the calcified bone matrix^10–13^. SWMR is sensitive to substances, which distort the magnetic field, like paramagnetic or diamagnetic substances. Bone minerals are diamagnetic and can therefore be visualised and differentiated from the surrounding tissue^14–17^. SWMR also enables a differentiation between tissue/substances with paramagnetic and diamagnetic properties^18–23^.

The aim of this study was to evaluate the potential of SWMR for angle measurements reflecting hip morphology, using radiographs as reference standard.

## Material and methods

### Study population

This prospective single center study was approved by and registered with the Ethics Committee of Charité University Medical Center Berlin. All methods were carried out in accordance with relevant guidelines and regulations for involving human participants in the study. All patients were informed and signed written consent*.* Patients with pain and clinically suspected pathologies of the hips, who had a clinical indication for MRI and radiograph, were clinically recruited and included in the study*.* Exclusion criteria were age < 18 years, not MR-compatible devices (e.g. pacemaker), pregnancy, breast-feeding, patients with mental disorders or who were unable to give consent.

### Imaging protocol

A 1.5 T scanner (Avanto, Siemens Medical Solutions, Erlangen, Germany) with a standard surface coil was used for all patients. All patients were examined with a standard hip protocol, including coronal T1 TSE images with the following imaging parameters: Distance factor 20%, phase coding direction right to left, phase-oversampling 100%, field-of-view 400 mm^2^, field-of-view phase 100%, matrix 448, TR/TE = 600/22 ms, 180 degree flip-angle, slice-thickness 4 mm, phase resolution 75%, phase partial fourier off, fat and water suppression off. Additionally, coronal SWMR was acquired in all patients, including magnitude images and reconstructed phase images^24–26^*.* SWMR includes SWMR magnitude images, deriving from a velocity-compensated 3D-GRE sequence. In addition, SWMR also includes the reconstruction of phase information^24–26^*.* For SWMR the following imaging parameters were used: Distance factor 20%, phase coding direction right to left, phase-oversampling 10%, field-of-view 320 mm^2^, field-of-view phase 100%, matrix 320, TR/TE = 49/20 ms, 15 degree flip-angle, slice-thickness 3 mm, phase resolution 100%, phase partial fourier off, fat and water suppression off*.* Magnitude images and phase images were automatically reconstructed^24^. Acquisition time of the SWMR sequence was 4:37 min*.* After the acquisition a coronal maximum intensity projection (SWMR MIP) of the magnitude and phase images was reconstructed with a thickness of 8 cm, overlap of 100% and a distance of 0 μm. If the femoral head, neck and acetabulum could not fully imaged on a slice-thickness of 8 cm, the thickness was chosen larger. A MIP with the same parameters was reconstructed from T1 TSE coronal images (T1w MIP). T2 images did not generate a contrast on the MIP images to analyze the angles.

Each patient additionally was examined with a radiograph in AP pelvic view either before or after the MRI.

### Imaging analysis

The image analysis was carried out on PACS workstations (Centricity Radiology RA1000, GE Healthcare, Little Chalfont, UK). One radiologists with more than 5 years (SMB) of diagnostic experience in musculoskeletal imaging reviewed all images twice independently in a randomized fashion. A second radiologist with more than 3 years (LCA) of diagnostic experience in musculoskeletal imaging reviewed a subgroup of 21 hip joints also independently in a randomized fashion. While evaluating the MR images, readers were blinded to the radiographs. On radiographs, coronal T1w MIP-images and coronal magnitude SWMR MIP-images, the lateral center–edge angle of Wiberg, Tönnis angle, Sharp´s angle and the caput-collum-diaphyseal angle were each measured. Tönnis angle was measured between a horizontal line and a line between the lateral and inferior end of the acetabular sourcil^1,5^. The lateral center–edge angle of Wiberg is the angle between a vertical line and a line through the center of the femoral head and the most lateral edge of the acetabulum^4^. The Sharp´s angle was measured between a line connecting the inferior aspect of the left and right acetabular tear drop and a line between the inferior aspect of the acetabular tear drop and the acetabular edge of each side^27^. The caput-collum-diaphyseal angle was measured between the longitudinal axis of the femoral neck and shaft^6^.

As a possible reason for different accurate results of angle measurements on T1w MIP and SWMR MIP, the signal intensity between the bone and the surrounding tissue could be assumed. To receive a quantitative value for the differentiation between bone and the surrounding soft tissue, the signal intensities of the bone and the surrounding tissue was measured on SWMR and T1w MIP-images. Regions of interest (ROIs) were placed in the femoral head, the gluteal muscles and the adductor muscles on both sides. For all ROIs, the largest common area of target tissue on SWMR MIP-images and T1w MIP-images was identified for each pair of images. The ROIs in the femoral head had a ellipsoid shape and covered the whole femoral head, excluding the rest of the bone and other tissues. ROIs of the gluteal muscles were drawn in a triangular shape. The lines of the triangular ROIs were drawn parallel to the iliac bone, the femoral neck and along the lateral subcutaneous fat tissue. The ROIs of the adductor muscles had a square shape. For this ROIs, two horizontal lines were drawn, one in line to the trochanter minor and the other one parallel to the caudal edge of the image. Afterwards, these lines were interconnected medial to the femoral bone and lateral to the medial subcutaneous fat tissue. When drawing the ROIs of the muscles, bones and subcutaneous fat tissue were excluded. Areas with strong artefacts, e.g. because of metal implants, were excluded of the selected regions. In each patient exactly the same ROIs were copied from one type of image weighting on to the same area in the other type of image weighting. A doublecheck was performed whether there were any violations of the above mentioned rules for outlining ROIs. Deviations were corrected on SWMR MIP-images and T1w MIP-images.

The ratio between the average intensity of the femoral head and the muscles was calculated and compared between SWMR MIP-images and T1w MIP-images.

### Statistical analysis

To determine the relationship between the measured angles on MR-sequences and radiographs, linear regression was applied. Bland–Altman plots were used to test if the size of the angles differ between MR-sequences and radiographs. To test intrareader and interreader variability between the measurements of the angles, linear regression and Bland–Altman plots were used. The smallest detectable difference (SDD) was calculated as a parameter for the reproducibility of the measurements between SWMR and radiographs.

Ratios of the femoral head/muscles on SWMR and T1w MIP-images were transformed with a natural logarithm and afterwards students t-test was used to calculate the difference between the ratios, using α < 0.05 for the level of significance.

## Results

### Patient population

From April 2014 to January 2019, 40 patients (23 males, mean age 44.04 ± 15.08 years, age range 19–71 years; 17 females, mean age 47.1 ± 21.3 years, age range 22–84 years) with pain and clinically suspected pathologies of the hips were referred to our department for imaging*.* 16 patients had deformities of the acetabulum or the femur like dysplastic hips or CAM/FAI impingement, in eight patients the most important diagnosis was a osteoarthritis, six patients had a avascular necrosis of the femoral head and in seven patients the most likely cause for the pain were pathologies of the soft tissue like muscles, tendons, bursae or the labrum. In two patients no morphological cause for the pain could be detected. One patient had to be excluded because of imaging artefacts on SWMR (strong movement artefacts of the intestines in the pelvis). 9 patients had an hip prosthesis or underwent a periacetabular osteotomy (PAO) of one hip, therefore in these patients, the angles of only one hip were measured. In the other 30 patients, both hips could be measured. Overall the angles of 69 hips were measured in this study. On the images of the patients after PAO or implantation of prosthesis, the acetabular tear drop of the side of the surgery could not be defined for certain, therefore Sharp´s angle was not measured on the other side in these patients. The mean time interval between MRI and radiograph of the patients was 89 days.

### Correlation of the center–edge angle of Wiberg

**Figure 1:**
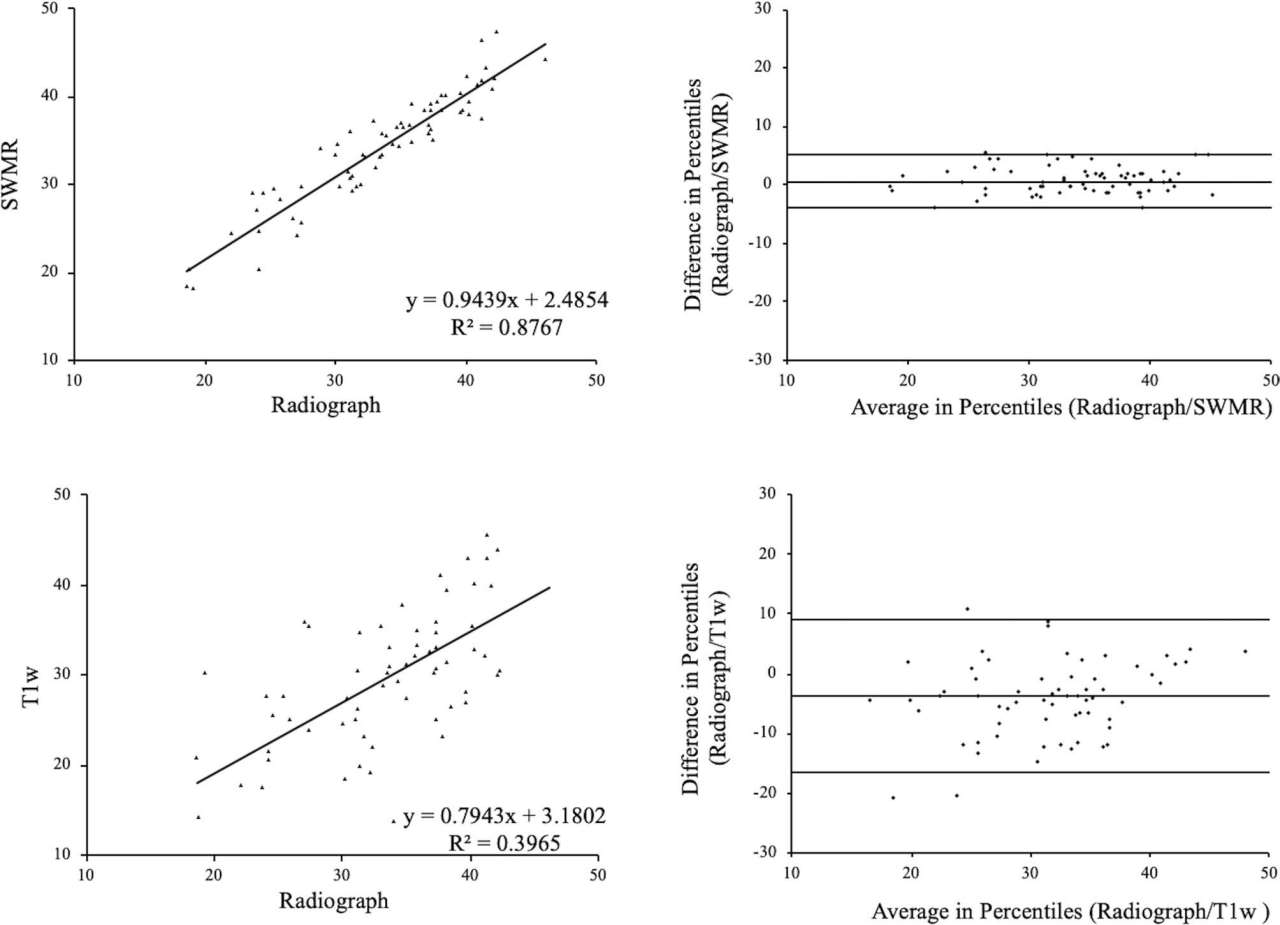
Center–edge angle of Wiberg. Linear regression and Bland–Altman plot of the center–edge angle of Wiberg on radiographs compared to susceptibility-weighted magnetic resonance imaging (SWMR) and T1-weighted magnetic resonance imaging (T1w). The measurements show a very strong correlation for the comparison between SWMR and radiographs (R^2^ = 0.88) and a moderate correlation for the comparison between T1w and radiographs (R^2^ = 0.40).

The center–edge angle of Wiberg showed a very strong correlation between SWMR MIP-images and radiographs (y = 0.94x + 2.49, R^2^ = 0.88, p < 0.05). Whereas T1w MIP-images showed a lower correlation (y = 0.79x + 3.18, R^2^ = 0.40, p < 0.05) (see Fig. 1). Intraobserver measurements correlated very strong on radiographs (y = 0.94x + 1.39, R^2^ = 0.86, p < 0.05) and SWMR MIP-images (y = 0.97x + 1.00, R^2^ = 0.91, p < 0.05) and strong on T1w MIP-images (y = 0.79x + 8.43, R^2^ = 0.61, p < 0.05).

The interobserver correlation showed a very strong agreement for SWMR (y = 0.98x + 1.23, R^2^ = 0.93, p < 0.05) and radiographs (y = 0.93x + 3.71, R^2^ = 0.82, p < 0.05) and a moderate agreement for T1w (y = 0.41x + 15.23, R^2^ = 0.31, p < 0.05).

### Correlation of Tönnis angle

**Figure 2:**
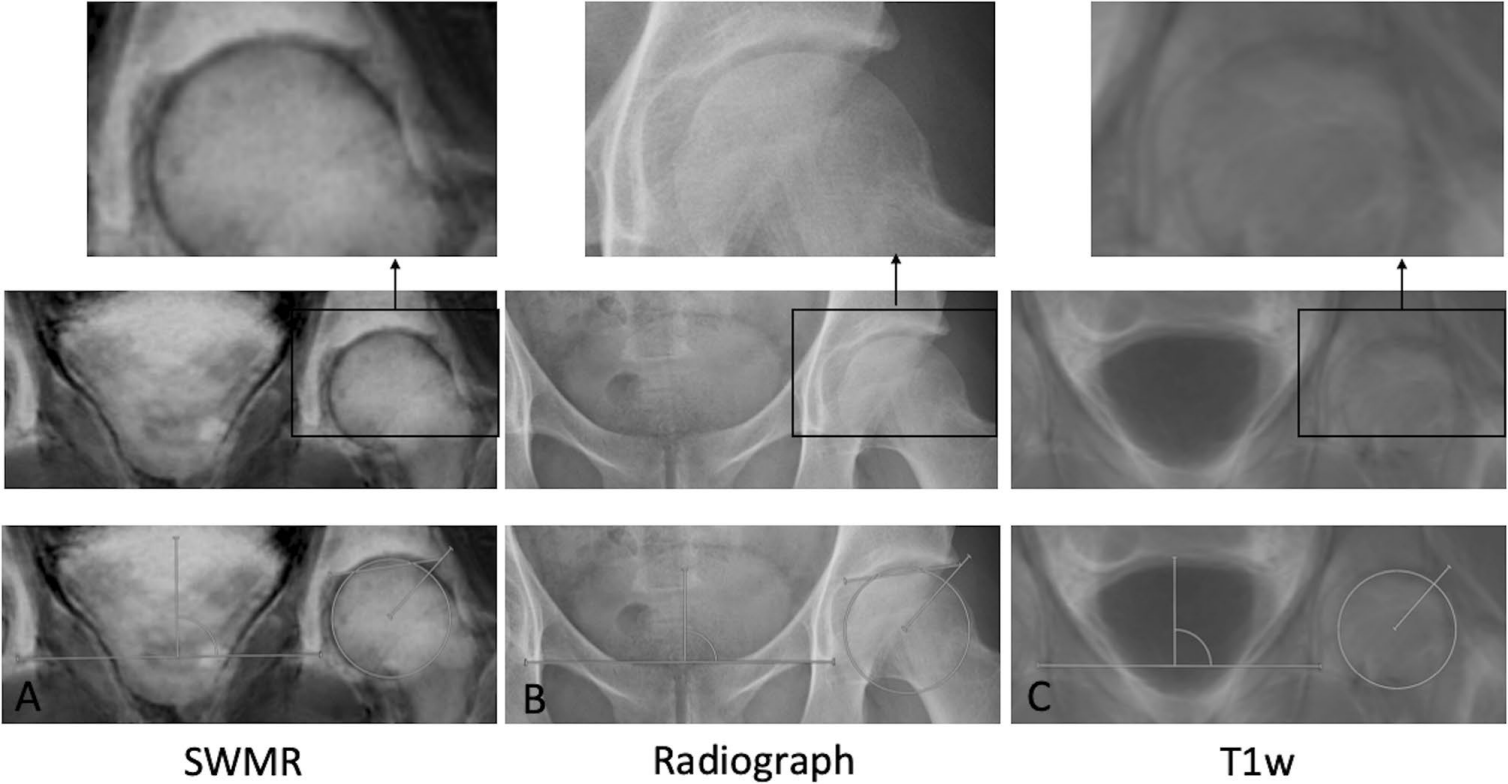
Example for angle measurements. Radiograph in anteroposterior view (**B**) and coronal maximum intensity projections of susceptibility-weighted MRI magnitude images (SWMR; **A**) and T1weighted MRI (T1w; **C**) with magnifications and angle measurements. Tönnis angle could not be measured on T1w because the acetabular sourcil could not be identified reliably.

Regarding the degree of Tönnis angle SWMR showed a very strong correlation with radiographs (y = 0.86x + 1.60, R^2^ = 0.86, p < 0.05). On T1w MIP-images, Tönnis angle could not be measured because the acetabular sourcil could not be identified (see Fig. 2). The intraobserver measurements also showed a very strong agreement on radiographs (y = 0.98x + 0.30, R^2^ = 0.89, p < 0.05) and on SWMR MIP-images (y = 0.91x + 0.70, R^2^ = 0.84, p < 0.05). As well they showed a very strong interobserver correlation (SWRM: y = 0.90x + 1.03, R^2^ = 0.95, p < 0.05; radiographs: y = 1.00x + 0.52, R^2^ = 0.86, p < 0.05).

### Correlation of Sharp´s angle

**Figure 3:**
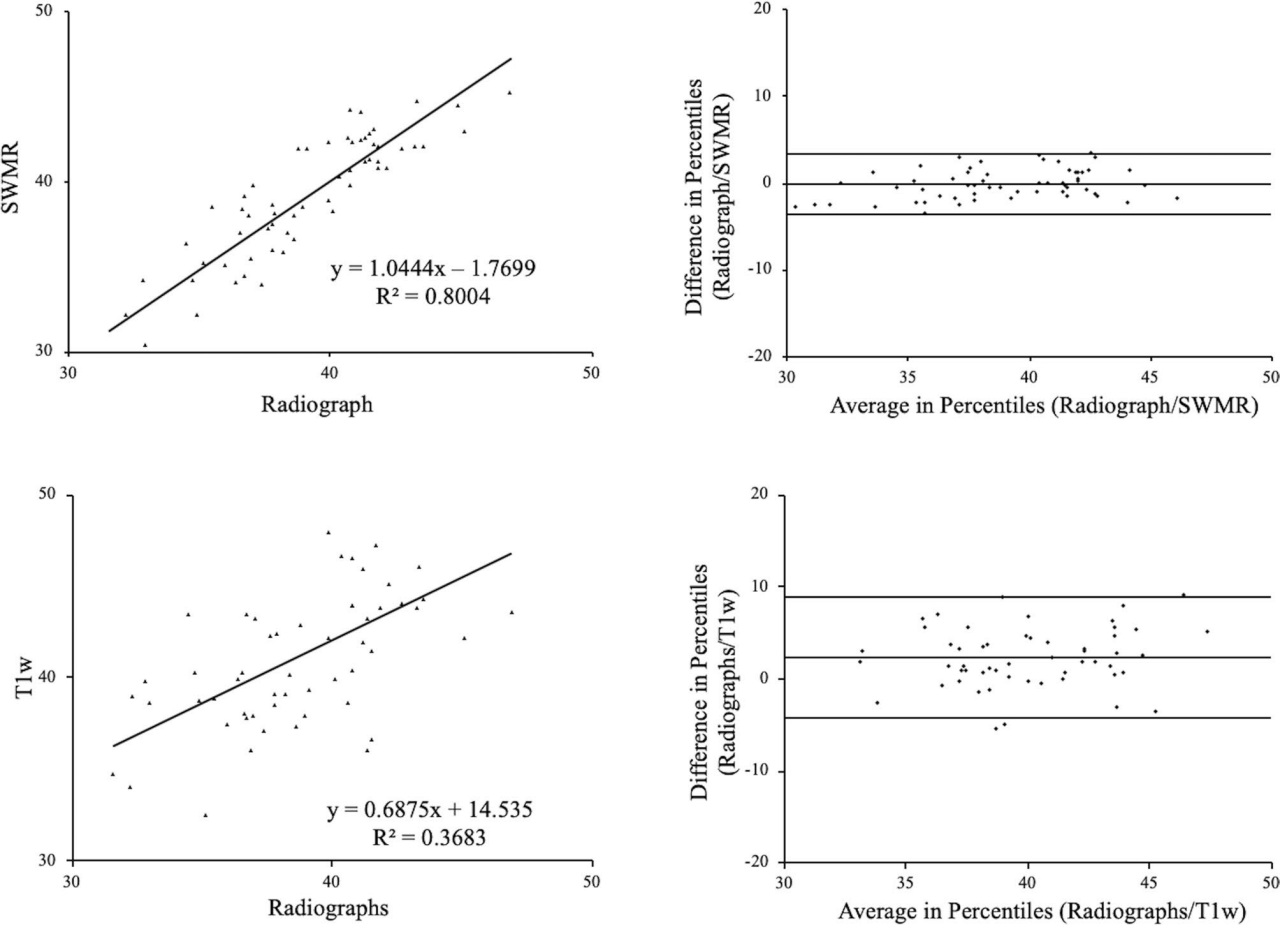
Sharp´s angle. Linear regression and Bland–Altman plot of Sharp´s angle on radiographs compared to susceptibility-weighted magnetic resonance imaging (SWMR) and T1-weighted magnetic resonance imaging (T1w). The measurements show a very strong correlation for the comparison between SWMR and radiographs (R^2^ = 0.80) and a moderate correlation for the comparison between T1w and radiographs (R^2^ = 0.37).

Sharps angle demonstrated a close correlation between SWMR MIP-images and radiographs (y = 1.04x + 1.77, R^2^ = 0.80, p < 0.05) but markedly lower correlation between T1w MIP-images and radiographs (y = 0.69x + 14.54, R^2^ = 0.37, p < 0.05) (see Fig. 3). Also radiographs and SWMR MIP-images showed a very close correlation regarding the intraobserver agreement (radiographs: y = 0.87x + 5.04, R^2^ = 0.87, p < 0.05; SWMR: y = 0.82x + 7.21, R^2^ = 0.75, p < 0.05) and a moderate correlation on T1w MIP-images (y = 0.49x + 20.16, R^2^ = 0.25, p < 0.05). The interobserver correlation for Sharps angle was very strong for SWMR (y = 0.76x + 9.52, R^2^ = 0.78, p < 0.05) and radiographs (y = 0.88x + 5.10, R^2^ = 0.85, p < 0.05) but weak for T1w (y = 0.07x + 39.51, R^2^ = 0.06, p < 0.05).

### Correlation of the caput-collum-diaphyseal angle

The caput-collum-diaphyseal angle showed a strong correlation between radiographs and SWMR MIP-images (y = 0.67x + 44.58, R^2^ = 0.38, p < 0.05) and a moderate correlation between T1w MIP-images (y = 0.71x + 35.18, R^2^ = 0.18, p < 0.05) and radiographs. The intrareader agreement was very strong for radiographs (y = 0.85x + 19.49, R^2^ = 0.80, p < 0.05), strong on SWMR MIP-images (y = 0.65x + 44.95, R^2^ = 0.54, p < 0.05) and moderate on T1w MIP-images (y = 1.134x + 21.03, R^2^ = 0.22, p < 0.05). Interobserver correlation was very strong for SWMR (y = 1.17x–22.42, R^2^ = 0.84, p < 0.05) and radiographs (y = 0.85x + 19.34, R^2^ = 0.85, p < 0.05), but moderate for T1w (y = 0.23x + 102.44, R^2^ = 0.31, p < 0.05).

### Smallest detectable difference

For the correlation of the angle measurements between radiographs and SWMR, the SDD with the sample size of 69 patients is R^2^ = 0.056. The calculated R^2^ between radiographs and SWMR was much higher than the SDD for all angles (R^2^ = 0.88/0.86/0.80/0.38). Therefore a good reproducibility of the measurement can be assumed.

### Assessment of the signal intensity between femoral head and muscles

**Figure 4:**
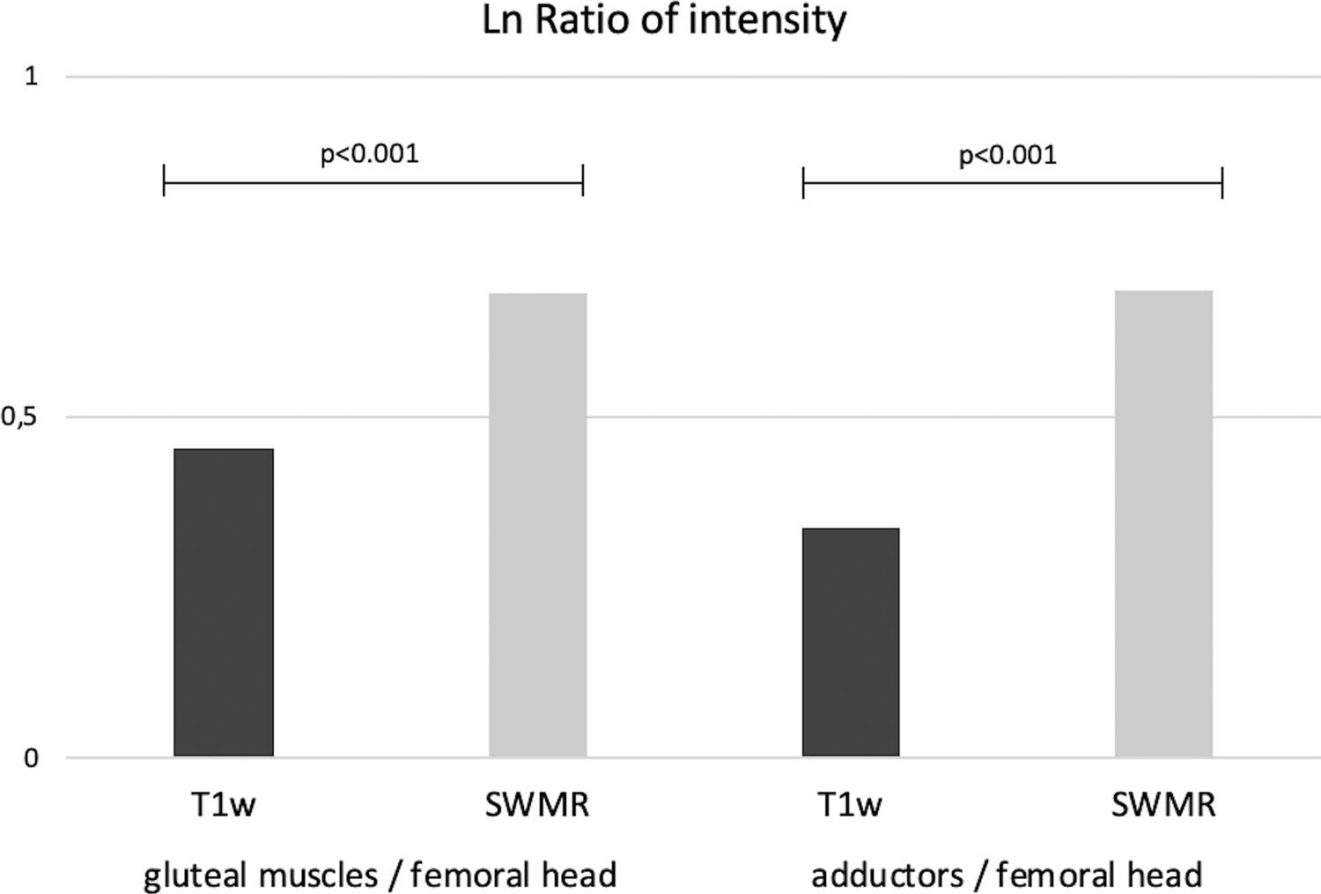
Ratio of intensity. Intensity was measured in the femoral head, the gluteal muscles and the adductors on coronal maximum intensity projections of susceptibility-weighted MR magnitude images (SWMR) and T1weighted MRI (T1w). The natural logarithm (Ln) of the ratio between the muscles and the femoral head was built and compared between SWMR and T1w, using students t-test. The ratio was significant higher on SWMR than on T1w for both muscles (p < 0.001).

**Figure 5:**
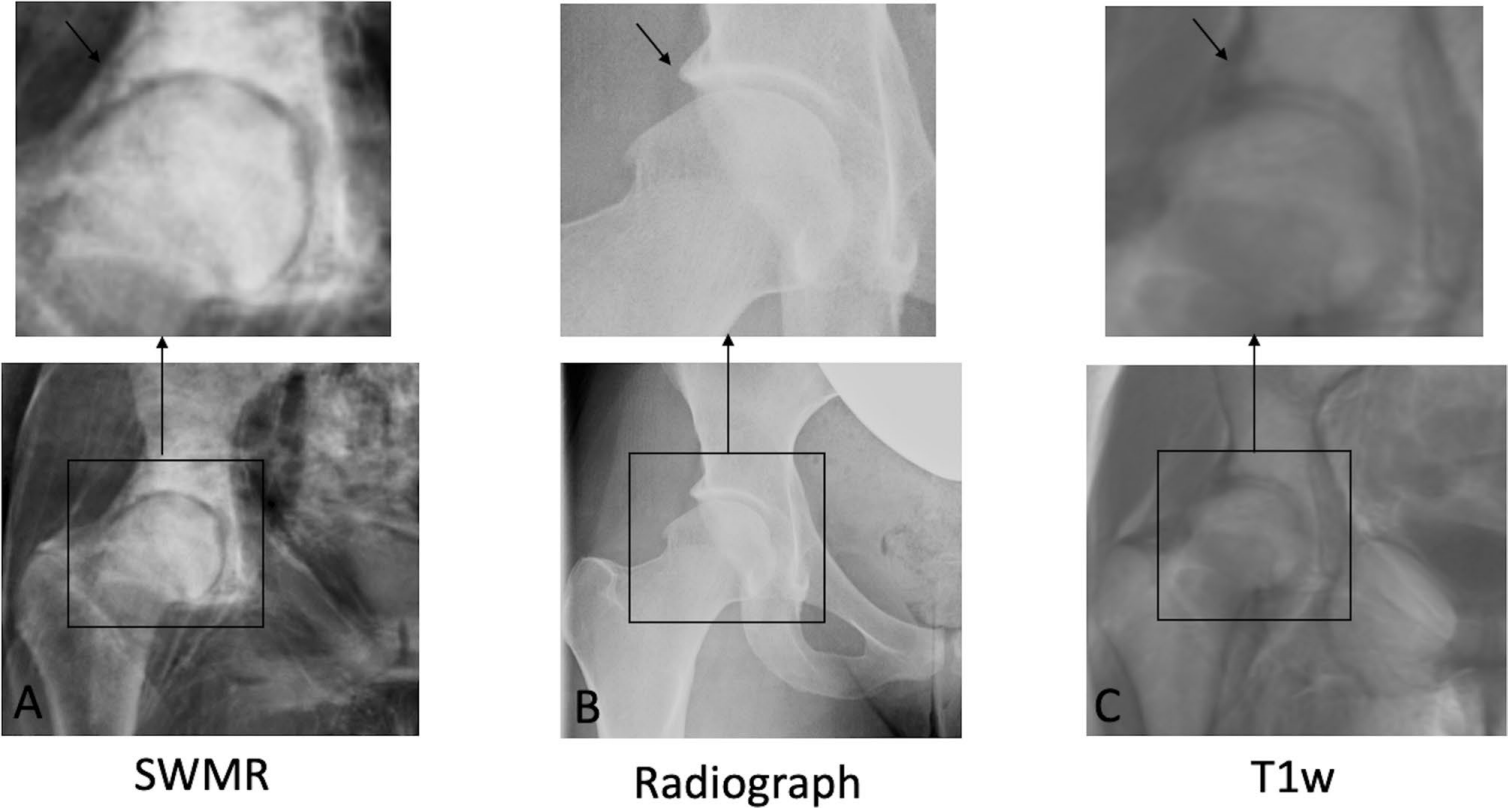
Visibility of the lateral edge of the acetabulum. Radiograph in anteroposterior view (**B**) and coronal maximum intensity projections of susceptibility-weighted magnetic resonance magnitude images (SWMR; **A**) and T1weighted magnetic resonance imaging (T1w; **C**) with magnifications. On SWMR and radiograph, the lateral edge of the acetabulum (arrow) can be identified sharp and clearly. On T1w, the identification of the lateral edge is more difficult and has a blurred shape.

The average of signal intensity of the femoral head was 59.8 for SWMR MIP-images and 261.63 for T1w MIP-images. In the muscles, an average of signal intensity of 118.11(gluteal) and 117.95 (adductors) was measured on SWMR MIP-images and 167.22 (gluteal) and 187.23 (adductors) on T1w MIP-images. The average of the ratio between the femoral head and the muscles was 2.00 (gluteal) and 2.02 (adductors) for SWMR MIP-images and 1.60 (gluteal) and 1.42 (adductors) on T1w MIP-images. The natural logarithms of the ratios were significant higher on SWMR than on T1w MIP-images (p < 0.0001) (see Fig. 4). Figure 5 shows an example for the imaging contrast of radiographs, SWMR and T1w MIP-images.

## Discussion

This study demonstrates that SWMR enables the reliable assessment of the bone morphological measurements of Sharp´s angle, Tönnis angle, lateral center–edge angle of Wiberg and caput-collum-diaphyseal angle compared to radiographs with a higher accuracy than conventional MR images. Patients with possible pathologies of the hips may benefit from the addition of SWMR to a standard MR imaging protocol, as it offers additional information regarding morphometric parameters of the osseous structures of the bone, without the need for radiation exposure. This could be especially relevant in a young and/or female patient population.

Imaging of the hip is important for the diagnosis of many diseases of the hip joint. In young adults, suspected deformities like dysplasia or femoroacetabular impingement and the resulting early degeneration of the hip are common reasons to perform an imaging examination. To assess the degree of the suspected deformity, the most morphometric parameters are measured on plain radiographs, e.g. the AP pelvic view. Specific protocols can also include additional other projections like frog leg or lateral views. In addition, MRI of the hip is performed in many cases. MRI is the method of choice to evaluate the bone marrow, the cartilage, the acetabular labrum and the surrounding soft-tissue^1^. Therefore early preradiographic signs of osteoarthritis can be visualised using MRI^3^.

In this study, different angles were measured on reconstructed SWMR and T1w MIP-images comparable to the measurement on AP pelvic views. Sharp´s angle, Tönnis angle and lateral center–edge angle of Wiberg showed a very strong correlation between SWMR MIP-images and radiographs with a very strong interobserver and intraobserver correlation. On T1w MIP-images, only a moderate to strong correlation to radiographs with a moderate to very strong intraobserver and interobserver correlation was measured. A potential reason for the limited performance was that the demarcation of the acetabular edge was challenging on T1w MIP-images, because the acetabular labrum and the cortical bone both appear hypointense on T1w-images. Additionally, the drawing of a horizontal line could not be performed reliably on T1w MIP-images because the inferior aspect of the acetabular teardrop was often difficult to identify.

In contrast, SWMR MIP-images could overcome these difficulties. SWMR is a 3-dimensional MRI gradient echo sequence, which highlights changes in magnetic susceptibility by generating contrasts based on differences in magnetic susceptibility. Magnetic susceptibility is the effect a substance generates, when it is placed in a magnetic field^14,24^. Each substance or tissue behaves different in a magnetic field and therefore leads to a specific distortion of the magnetic field. Paramagnetic substances (e.g. ferritin and deoxyhaemoglobin) line up with the external magnetic field, whereas diamagnetic substances (e.g. calcium and bone minerals) line opposed to the external magnetic field^14,24,28,29^. In different studies, it was shown that SWMR enables the differentiation between paramagnetic and diamagnetic substances based on their phase shift^16,22,28,30–34^. In this study, SWMR enabled the identification of the osseous measuring points for a reliable measurement of Sharp´s angle, Tönnis angle and lateral center–edge angle of Wiberg.

The main reason for the better performance of the SWMR sequence was that the contrast between the bone and the surrounding soft tissue is substantially higher on SWMR MIP compared to T1w MIP-images. The ratio between the femoral head and the surrounding muscles was also substantially higher on SWMR than on T1w sequences.

The measurement of the CCD-angle showed a lower correlation for SWMR and T1w MIP-images than the other measured angles. A possible explanation for this result are the artefacts on the borders of the SWMR and T1w-images.

In a previous study, Stenzender et al. already showed, that MRI provides similar morphometric measurements for the most hip parameters^9^. They used three dimensional unilateral isotropic true-FISP sequence and reconstructed them in different straight coronal positions with a slice thickness of 0.63 mm. In our opinion the advantage of bilateral MIP-images is, that the bilateral reconstruction enables the identification of the acetabular teardrop and therefore a reliable measurement to the body axis and the measurement of Sharp´s angle. In Addition, it might be easier and more reproducible in clinical routine to reconstruct MIP images and have the same visual impression like an AP pelvic view. Therefore established measuring methods can be proceeded as usual by the reader.

In this study, only adults were included. It remains unclear if SWMR also enables the reliable measurement in skeletal immature individuals. The results of this study cannot directly be transferred to field strength other than 1.5 T because susceptibility relies on the magnetic field strength. In 3 T or 7 T scanners, susceptibility is stronger than on 1.5 T and it remains unclear, if SWMR has the same value to evaluate morphometric parameters of the hip on that field strengths. Metal implants cause strong susceptibility artefacts, especially in SWMR, with the consequence that hips with prosthesis cannot be evaluated. Our data is not applicable to patients with contraindications to MRI. Further studies with a bigger study population and additional morphometric measurements are needed to verify the value of SWMR reflecting the overall hip morphology.

In conclusion, SWMR enables the reliable assessment of Sharp´s angle, Tönnis angle, lateral center–edge angle of Wiberg and caput-collum-diaphyseal angle compared to radiographs with a higher accuracy than conventional MR images. Patients with suspected abnormalities of hip morphology may benefit from the addition of SWMR to a standard MR imaging protocol, as it offers additional information, without radiation exposure.

## Data Availability

All relevant data analysed or generated in this study are included in this published article.
