# Author Correction: Value of susceptibility‑weighted imaging for the assessment of angle measurements reflecting hip morphology

**DOI:** 10.1038/s41598-021-96788-5

**Published:** 2021-08-27

**Authors:** Sarah M. Böker, Lisa C. Adams, Ute Lina Fahlenkamp, Gerd Diederichs, Bernd Hamm, Marcus R. Makowski

**Affiliations:** 1 Department of Radiology, Charité, Charitéplatz 1, Berlin, Germany; 2 Department of Radiology, Technical University of Munich, Ismaninger Str. 22, 81675 Munich, Germany; 3 King’s College London, School of Biomedical Engineering and Imaging Sciences, United Kingdom, St Thomas’ Hospital Westminster Bridge Road, London, SE1 7EH UK

Correction to: Scientific Reports 10.1038/s41598-020-77671-1, published online 01 December 2020

The original version of this Article omitted affiliations for Marcus R. Makowski. The correct affiliations are listed below

Department of Radiology, Charité, Charitéplatz 1, Berlin, Germany

Department of Radiology, Technical University of Munich, Ismaninger Str. 22, 81675, Munich, Germany

King’s College London, School of Biomedical Engineering and Imaging Sciences, United Kingdom, St Thomas’ Hospital Westminster Bridge Road, London, SE1 7EH, UK

The original Article has been corrected.

